# Supplementary material for: Splice-Junction-Based Mapping of Alternative Isoforms in the Human Proteome
Source: Cell Rep. Author manuscript; Available in PMC 2020 Jan 15. (PMC6961840; doi:10.1016/j.celrep.2019.11.026)
Supplement: 3 [file NIHMS1546469-supplement-3.zip › DF2/PXD0010154/Thyroid-23-P01266-LQASGAGTWCVDPASGEELRPGSSSSAQCPR.pdf]

A

Predicted sequence disorder and sequence features of P01266

Peptide: LQASGAGTWCVDPASGEELRPGSSSSAQCPR Junction: sp|P01266|THYG_HUMAN|ENSG00000042832|SE2|49953|chr8|132900339|132901553|+0|r4|T1 TrNovel: TRUE

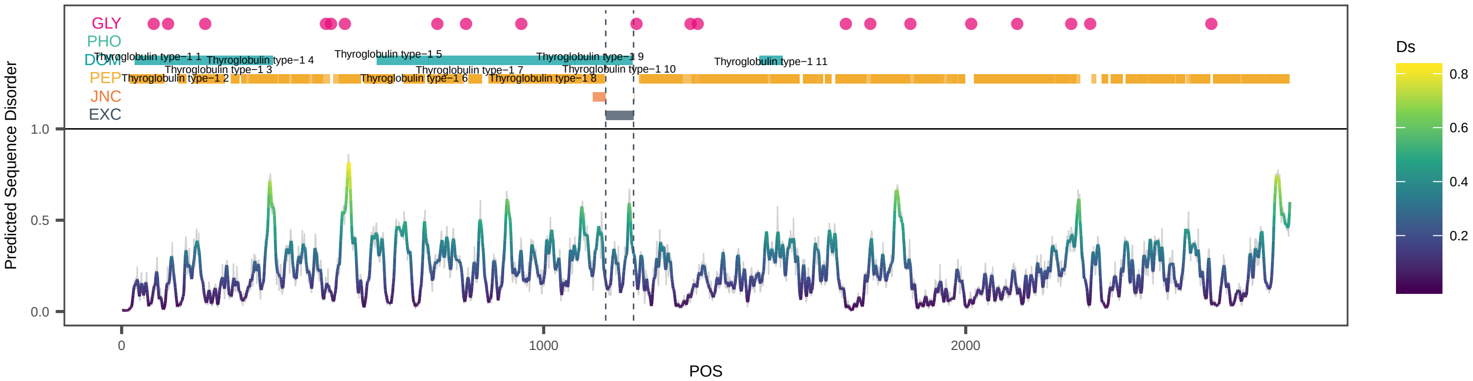

B

Distribution of sequence disorder in excised vs. mapped and non−excised regions of protein

M−W P−value vs. mapped: 0.00242 vs. non−excised: 0.00141

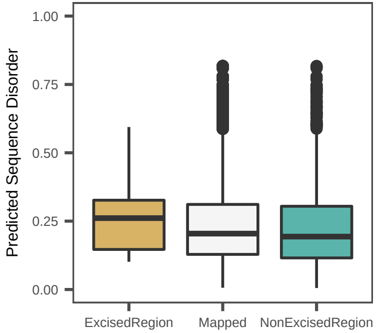

C
